# A historical dataset of federal government spending changes for Canada

**DOI:** 10.1016/j.dib.2022.108850

**Published:** 2022-12-24

**Authors:** Syed Muhammad Hussain, Lin Liu

**Affiliations:** a James Madison University, College of Business, Zane Showker Hall, 421 Bluestone Dr, Harrisonburg, VA, 22807, USA; b University of Liverpool, Management School, Chatham Building, Chatham Street, L69 7ZH, Liverpool, UK

**Keywords:** Narrative approach, Exogenous and endogenous spending changes, Fiscal policy, Canada, Budget documents

## Abstract

This dataset presents a narrative record of all announced federal government spending changes in Canada between 1949q1 and 2012q1. We use the federal government's budget documents, mostly the budget speech, to document announced spending measures. Other budget documents that we use include the Economic Statements, Financial Statements, Mini Budgets, Interim Budgets, and Economic and Budget Updates. We document the motivation behind each announced measure. Based on these motivations, we classify spending changes as exogenous or endogenous. Exogenous changes are those that are not motivated by contemporaneous economic conditions of the country. Endogenous changes are those that are taken in response to current economic conditions. We also document the size of a change, whether it was intended to be temporary or permanent, the duration of the measure, and the size of the measure that was to be implemented in the same year as it is announced. This is the first dataset for any country that comprehensively presents all spending changes undertaken by a government.

**Specifications Table**

**Table:** 

Subject	Economics.
Specific subject area	Macroeconomics, Public Economics
Type of data	Excel Files, Text Document
How the data were acquired	Data created by reading all budget documents – including the budget speeches, financial statements, economic statements, interim budgets, and economic and financial updates. Each spending change announced by the Federal government was documented using these documents.
Data format	Raw, Processed
Description of data collection	The House of Commons debates containing the budget speeches were studied to create the dataset on all announced spending changes. For some years, other budget documents were also consulted.
Data source location	The sources used to collect the data include different types of budget documents of the Government of Canada. The accompanying document cites the page number of the budget speech from where information on each spending change was collected.
Data accessibility	Repository name: Mendeley Data DOI: https://doi.org/10.17632/7k8rbvksvk.1 Direct URL to the dataset: https://data.mendeley.com/datasets/7k8rbvksvk/1
Related research article	Hussain, Syed M. and Liu, Lin, Macroeconomic Effects of Government Spending Shocks: New Narrative Evidence from Canada, Journal of Macroeconomics. In Press. https://doi.org/10.1016/j.jmacro.2022.103483

## Value of the Data

• Despite the importance of fiscal policy, there is little consensus among economists about the effects of fiscal policy. The challenge in estimating the effects of fiscal policy is to identify exogenous spending changes – those that are uncorrelated with the current state of the economy. This dataset documents all announced government spending changes for Canada for the time period 1949q1-2012q1 and identifies exogenous spending changes using the motivation behind each change.
• Existing empirical techniques used to identify exogenous changes in government spending fail to capture anticipation effects – that spending changes are often announced in advance of their implementation. Anticipated spending changes can have different effects from unanticipated changes, so it is important to construct a measure of *news* about upcoming changes in spending [1]. This dataset uses the announcements about spending changes to construct a novel variable that captures the news about upcoming changes in government spending.
• The dataset can be used to study how the spending policy of the Federal government has evolved over time and how the government has responded to various types of changes at different points in time.
• The dataset can be used to study the macroeconomic effects of changes in government spending. It can also be used to study the effect of spending changes on disaggregated measures of the economy like income inequality and industrial production of specific sectors.
• The methodology to identify exogenous government spending changes used in this dataset can be used for other countries to construct similar datasets.

## 1 Objective

The question of macroeconomic effects of government spending has garnered renewed interest in the aftermath of the great recession of 2008 and the ongoing COVID-19 induced economic crises. Despite the importance of this question, there is a surprising lack of consensus about the effects of government spending changes. The study of macroeconomic effects of government spending changes becomes complicated because of the problem of simultaneity – changes in government spending cause output to change but at the same time output causes government spending to change.

Literature has tackled this identification problem in two ways. First, several studies have used econometric techniques to identify exogenous changes in government spending. Prominent studies in this strand of literature include Blanchard and Perrotti [2] who use a Cholesky decomposition in a structural vector autoregression model (SVAR) to achieve identification. However, Ramey [1] shows that identified spending shocks identified through SVAR techniques are in fact anticipated and hence do not accurately measure the effects of spending changes. Ramey [1] argues in favor of a narrative approach to identify spending shocks. In this approach, news about upcoming changes in government spending is recorded through several sources (like newspapers) and used to identify exogenous changes in government spending. Ramey [1], for example, uses news about military spending changes to identify exogenous government spending changes.

In this paper, we construct a new measure of news about *all* government spending changes for Canada for the period 1949q1-2012q1. Our measure is different from the measures used in the literature so far because we incorporate news about all spending changes rather than just military spending changes. Also, our measure is the first of its kind for any country other than the US.

Other studies have used the narrative approach to construct measures of tax changes for various countries. Romer and Romer [3] use historical records like Presidential speeches and Congressional Budget documents to document tax changes for the US for the post World War-II period. They use the motivations provided in the official documents to identify exogenous tax changes. Cloyne [4] uses budget speeches and Financial Statements to construct a similar measure for the UK. Hayo and Uhl [5] and Gil et al. [6] have constructed similar measures of Germany and Spain respectively.

Our measure of news about exogenous government spending changes combines the methodologies of Ramey [1] and Romer and Romer (2011). We follow Romer and Romer [3] and Cloyne [4] by using the motivation provided for each spending change to classify it as exogenous or endogenous. Endogenous changes are those that are made in response to contemporaneous happenings in the economy while exogenous changes are those that are orthogonal to the current state of the economy.

## 2 Data Description

The dataset consists of two files. The excel file, “Dataset submitted Canada Government Spending”, contains raw and processed data on spending changes for Canada for the period 1949q1-2012q1. Within this file, the sheet “Detailed Data Canada” contains all the spending changes that we documented for the sample period. We read all budget documents to identify government spending changes and then classified them as exogenous or endogenous based on the motivation behind them (the details of the approach used are presented in the next section).

The raw data on spending changes was used to calculate the present discounted values of all exogenous changes announced in a budget. For this, we used the duration of a spending change, together with the quarterly interest rate on 1–3-year bonds of Government of Canada. The quarterly interest rates were calculated as the average monthly interest rates on these bonds. We calculated four measures of present discounted values. They differ in the number of years we used for permanent changes to calculate their present values. These present discounted values correspond to the *news* about upcoming spending changes. Table 1 described the variables presented in this file

**Table 1:** Description of Variables in “Detailed Data Canada”.

Variable	Type	Description
Date	Numeric	Date of budget speech
Type of Budget	Character	Type of budget document (budget speech, budget papers etc)
Measure	Character	Brief description of each spending measure
Size	Numeric	The size of each spending measure
Note	Character	Information about any measure with non-standard spending pattern. E.g. if a spending change was announced in one year but was to be implemented in the future.
Classification	Character	Exogenous or Endogenous
Sub-classification	Character	Exogenous changes were divided into Ideological, Military, and Long-Run Changes.
Endogenous changes divided into Supply Stimulus and Demand Management changes.		
Type	Character	Permanent or Temporary
Duration	Numeric	Duration of temporary spending changes
One Year Size	Numeric	Size of measure announced and implemented in the same year
Transfer Payment	Numeric	1 if the measure is a transfer payment. 0 otherwise.
Interest Rate	Numeric	Quarterly interest rate on 1–3-year bonds of Government of Canada
PDV 1	Numeric	Present Discounted Value of announced changes
Assumption: permanent changes last for one year		
PDV 5	Numeric	Present Discounted Value of announced changes
Assumption: permanent changes last for five years		
PDV 10	Numeric	Present Discounted Value of announced changes
Assumption: permanent changes last for 10 years		
PDV I	Numeric	Present Discounted Value of announced changes
Assumption: permanent changes last forever.		

The processed data is presented in the sheet, “Government Spending Shocks”. It presents quarterly data on news about upcoming changes in government spending. To make values across different years comparable, we present the present discounted values as a fraction of the nominal Gross Domestic Product (GDP) of the previous quarter. The data file contains the variables that use the PDV 5 variable from “Detailed Data Canada”. We assign each observation to a quarter using the dating assumption of Romer and Romer [3]. If a spending change was announced in the second half of the quarter, we assign it to the following quarter.

The sheet also presents a measure of spending changes that were announced and implemented in the same year. We call this series ‘Announced and Implemented Changes’. This series uses the ‘One Year Shock’ from the file “Detailed Data Canada”. Like the news variables, we divide this series by GDP of the previous quarter as well. Table 2 summarizes the variables found in “Government Spending Shocks”.

**Table 2:** Description of Variables in “Government Spending Shocks”.

Variable	Type	Description
Date	Numeric	Quarter in which spending change was announced
Exogenous News	Numeric	Sum of PDV 5 of exogenous spending changes announced in a budget divided by nominal GDP of previous quarter.
Endogenous News	Numeric	Sum of PDV 5 of endogenous spending changes announced in a budget divided by nominal GDP of previous quarter.
Announced and Implemented Changes	Numeric	Sum of sizes of those changes that are announced and implemented in the same year.

The second file in the dataset contains a narrative detail of all spending changes used in the dataset. This file is called “Narrative Record”. For each measure, the file presents several details (presented in the next section) and the sources from where the information was collected. In particular, each detail of a measure is followed by the page number of the document from where the information was collected.

## 4 Experimental Design, Materials and Methods

Our main source of information while constructing news about government spending changes is the budget speech. The budget speech is often the first time the government announces its fiscal plan for the upcoming year. A particular feature of the Canadian budget process – budget secrecy, mutes concerns about economy having prior knowledge of the changes to be announced. Budget secrecy is a long-standing tradition of the Canadian budget process whereby the contents of the upcoming budget are only known to the finance minister and members of the cabinet.

To construct our measure of news about upcoming government spending changes, we read all the budget speeches and record announced government spending changes. For most years in our sample, we relied upon the budget speech to gather all the required information. For some years, especially towards the end of the sample, the budget speech became less informative about the specific spending changes and hence we relied upon other budget documents, like the budget report, to document all spending changes.

For each budget, we document the following aspects

(1) Overall Context: We begin description of each budget by summarizing the overall context of the speech. This would often be found in the beginning of the speech and would set the tone for the spending changes to be made. For example, during the mid-1980’s, the government of Canada became concerned about the long-run debt situation of the economy. The budget speeches would typically begin by summarizing the state of the economy and then describing the concern of the government about the debt. Indeed, most of the changes announced in these budget speeches were designed to reduce the debt level of the economy.
(2) The Measure: We then begin describing the spending measures announced. First, we briefly describe the change that is announced.
(3) Reason: Next, we briefly describe the reason behind the change. This typically would come from the budget speeches. In most cases, it was easy to identify the reason behind an announced change and hence we keep this part brief.
(4) Size: Next, we document the size of the measure. We get this information mainly from the budget speeches but for some years, we consulted other documents like the Budget Plan or the Budget Papers to get this information as well.
(5) Status: We then document whether a change was intended to be permanent or not. In some cases, the information in the budget documents would make it explicitly clear whether a change was intended to be permanent or temporary. In other cases, we had to rely upon the description of the change and the language used in the budget documents to identify its status. For example, if the finance minister used terms like “per year” (e.g., spending on infrastructure would increase by $20 million per year) then we regard it as a permanent change. On the other hand, if the language used included specific number of years, then we regard it as temporary. The overall context of the budget speech would also help us in identifying the status of the change. For example, changes made in response to downturns in the economy were almost always temporary while most changes made in times of economic growth tended to be permanent.
(6) Classification: Having documented all the details about a particular change from the budget documents, we then classify it as endogenous or exogenous. Endogenous changes are those that are made in response to contemporaneous happening in the economy. These typically included those changes that were designed to offset the negative effects of a recession. Endogenous changes were further sub-classified as demand management and supply stimulus changes. Demand management changes were designed to boost aggregate demand of the economy whereas supply stimulus changes were designed to aid producers.

Exogenous changes were those that were uncorrelated with contemporaneous events of the economy. These were further classified into four categories. First, long-run spending changes were aimed at boosting the long-run growth of the economy. Second, spending changes could be motivated by ideological reasons and were aimed at improving efficiency and fairness in the economy. Third, deficit consolidation changes were designed to reduce long-run debt of the economy. Finally, military spending changes were during times of war or due to changes in the security situation of the country.
In most cases, the measure and the reason behind the measure made it clear why we chose a particular classification. In some cases where it was not clear, we added a brief explanation about our choice of classification.

Using our methodology, we ended up with 342 individual government spending changes for our sample period that was 1949:1-2012:1. Out of these, 71 were endogenous while the rest were exogenous.

## Ethics Statements

The study did not include work with human subjects or animals. It also did not involve data collected from social media platforms.

## CRediT Author Statement

**Syed Muhammad Hussain:** Conceptualization, Methodology, Investigation, Writing – Original Draft; **Lin Liu:** Conceptualization, Methodology, Writing – Review and Editing, Visualization.

## Declaration of Competing Interest

The authors declare that they have no known competing financial interests or personal relationships that could have appeared to influence the work reported in this paper.

## Data Availability

A Historical Dataset of Federal Government Spending Changes for Canada (Original data) (github)
